# Mr. Dawson, on Mercury in Venereal Affections

**Published:** 1805-06-01

**Authors:** G. P. Dawson

**Affiliations:** Sunderland


### *To the Editors of the Medical and Physical Journal.*

Gentlemen,

THE ingenious Mr. Cuming, to whose valuable communications the medical world is much indebted, has, in a concise and well-written paper, on the "Use of Mercury in Venereal Affections," so compleatly delivered my sentiments, as to leave but little for me to say on the subject, except that of assuring him of my hearty concurrence in all he has advanced, and how often I have lamented what he has now so well expressed and commented on. I have ever been of opinion, that mercury, for the cure of Syphilis, is often rashly ordered, and inconsiderately persisted in, under peculiar circumstances, which, judiciously considered, should forbid its use, and be superseded by more appropriate remedies. In every consideration respecting the use of mercury, but one, Mr. Cuming has most ably anticipated me; and I feel greater pleasure in referring the reader to his truly-excellent observations, than to any of my own. The one he has unnoticed is, the use of this medicine during the inflammatory stage of that disease. On this subject I beg leave to offer a few remarks. Before I commence, I request to be indulged while, in order to prevent unnecessary quibbling about words or terms, I explain what I mean by the "inflammatory stage." The appellation may or may not be proper; I use it, however, simply to express the inflamed state of a venereal bubo, or of a venereal node; and when, in the course of this paper, I use the word inflammation, I beg to be understood that I consider it in that light and in no other.

Whenever the venereal disease is attended with inflammation, I believe the exhibition of mercury to be hurtful and injudicious; and I have no hesitation in saying, that,

 in in all such cases, the use of this powerful medicine should be suspended, and not had recourse to, until that inflammation is subdued by antiphlogistic remedies. Mercury acts as an irritant, and cures the disease by exciting irritation and fever; and though it will remove a venereal action, yet was it never known to lessen an inflammatory one; exhibited then where inflammation prevails, it can only increase it, and exasperate the disease; yet what so common, as to see practitioners freely using mercury in irritable people labouring under a highly-inflamed bubo or node? or who has not seen the same practice pursued in dangerous cases of phymosis and paraphymosis? Why is it we so repeatedly see suppurated, ulcerated, and phagadenic buboes? The answer to this query is simple: the consequence of the imprudent use of mercury, resolutely continued, in spite of excessive debility, high inflammation, threatened suppuration, or indeed any other impending evil, clinging to it, as if it were a sheet anchor on which all their dearest hopes rested! In the incipient state of a bubo, or a node, the administration of mercury is vindicable and proper, and, where no untoward symptoms present themselves, its use should be persevered in as long as may be deemed requisite; but if, on the other hand, the tumour becomes painful and inflamed, then should mercury be laid aside, and not again resumed until the inflammation has been removed by proper means; for what so likely to increase inflammation, and produce suppuration, as mercury, a medicine highly irritable? or what can have a worse effect, in occasioning an unfavourable disposition in a suppurating sore, than mercury? Mercury then, whenever inflammation appears in a bubo, should not be used. If we succeed in discussing this inflammation by antiphlogistic means, it is well; again we may resort to a guarded exhibition of this remedy; but if we fail in this, and see suppuration approaching, still we forbear from mercury; we hasten this process by warm applications; we make an early opening into the part; we continue them till all inflammation has left the sore; then, and not before, we return to mercury, and continue it according to circumstances. A celebrated physician, while condemning the free and unguarded use of port wine in uterine hæmorrhages, says, "no matter whether the pulse is a menorrhagic or a weakened one; still red wine is indiscriminately given." With much propriety may we parody upon this, while speaking of the use of mercury in venereal affections: no matter whether the disease be attended with inflammation, debility, or any other unpleasant pleasant circumstance; still mercury is indiscriminately administered.

Some may cavil at what I write, and urge, in opposition to what I have asserted, that they have seen patients quickly recover under the practice I am now condemning. This I will not deny. On the contrary, I have frequently seen it myself; but, be it remembered, they did not receive their cure by good, but in spite of maltreatment. I might adduce many cases, if indeed any were necessary, to prove the superiority of the practice I am now recommending; the relation of one shall at present suffice:

A gentleman applied to me last winter, affected with a large chancre at the extremity of the urethra, accompanied with a bubo, considerably inflamed, and bearing indistinct marks of suppuration. The routine of practice I had been witness to in the early part of my life, would have induced me to have had immediate recourse to mercury; this plan of treatment, however, I did not pursue; I advised the black wash to be applied to the chancre, the application of warm poultices to the swelling, and a purge the succeeding morning. In three days the bubo was opened with a lancet; in three more the poultices were discontinued, and the tumour free from inflammation. Now I began with mercury; at this time the chancre was partly healed by the application of the calomel wash, and in less than three weeks he was compleatly well; nor was there the least vestige of the bubo remaining, except the scar made by the lancet. Had I in this case given mercury, in the first instance, I question much whether the cure would have been so speedy.

Speaking of mercury, it may not be foreign to the present subject, to consider what form of it is best adapted for the cure of syphilis. From observation, I am inclined to yield the palm of preference to mercurial frictions; this I conceive to be a safer and more accurate way of conveying mercury into the system than any other. The addition of a little camphor to the ointment I would advise, as tending much to facilitate absorption. Next to mercurial frictions, in my estimation, stands the blue pill. Other forms of mercury there are; but these are more or less doubtful: among the principal of these are, corrosive sublimate and calomel. Corrosive sublimate, though less certain than frictions or the blue pill, is yet a valuable medicine, particularly when it is necessary to saturate the constitution with mercury speedily, as in deep corroding ulcers, or venereal blotches; and in venereal ophthalmia, I have been assured it is of infinite service. Calomel is a violent and unsafe remedy, and, in my opinion, not to be relied on for the cure of this disease. It has fallen to my lot to have seen this medicine repeatedly used in venereal complaints; sorry am I to say, often unsuccessfully. I have not unfrequently seen secondary symptoms in the course of a few months succeed primary ones, after a supposed cure by calomel.

It has been dogmatically decreed, by very high authority, that lues venerea is never attended with constitutional action. On this point there still remains great diversity of opinion, and probably this long-debated question may never be compleatly decided. From a minute retrospect of this disease, one would, upon a review of certain circumstances, be induced to dissent from this opinion, and believe syphilis to be really attended with constitutional action. This idea receives considerable strength from observation; for in the intermediate time of primary and secondary symptoms, it is no very unusual thing to see patients labouring under constitutional ailment, without knowing what cause to attribute it to. A singular case of lues venerea, which happened within the sphere of my own knowledge, in my mind strongly corroborates this idea.

A gentleman, who had formerly been the subject of chancre, but of which he believed himself perfectly cured, suddenly began to lose flesh, and find his general health gradually impair, with slight attacks of fever in the evening. Unconscious of the cause, he sought no advice, and thus went on for a few months, evidently sinking under ill health, with regular febrile attacks. It happened that he was under the necessity of performing a long journey in a carriage, where, being much exposed to night air, he caught cold, and found considerable uneasiness in his throat. This attracted attention: and on examination, upon his arrival at an inn, he, to his inexpressible surprise, discovered a large venereal ulcer, which had destroyed nearly one half of the tonsil gland. This explained the true nature of his complaint, and in due time mercury effected a cure. Here then is an instance of lues venerea preceded, for months, by constitutional action! I must confess, I should like to know if Mr. Cuming's ideas coincide with mine, respecting the use of mercury in those cases of the venereal disease attended with inflammation.

Though, Gentlemen, I do not conceive gonorrhœa to be venereal, or to require mercury for its cure, yet it may not be superfluous briefly to recite a curious case of one, which lately happened under my care; curious, as it is a proof of the disease occuring six weeks and three days *post coitu.*

A young man, of an indolent though healthy constitution, had connection with a female, on the 27th of December, 1804, and, on the 10th of February, 1805, he became the subject of a virulent gonorrhæa, denoted by intense ardor urinæ, excessive discharge, and violent chordee. During the intermediate time, he had no intercourse with the sex whatever. Of this I am as certain, as if it had occurred in my own person. He had been thrice before the subject of the same disease; each of which appeared in the course of twelve days.

I am, &c.

*Sunderland, May* 7, 1805. G. P. DAWSON.

